# Supplementary material for: A UMLS-based spell checker for natural language processing in vaccine safety
Source: BMC Med Inform Decis Mak. 2007 Feb 12;7:3. doi: 10.1186/1472-6947-7-3 (PMC1805499; doi:10.1186/1472-6947-7-3)
Supplement: Additional file 13 — RAP application directory. Sets up directory for RAP (RDF application for PHP) source code [file 1472-6947-7-3-S13.gz › rap/doc/phpdoc/infModel/InfModel.html]

xml version="1.0" encoding="iso-8859-1"?


Docs For Class InfModel


## Class InfModel

Description

Description |
Descendents
| Methods (details)

A InfModel Model extends a MemModel , by adding the ability to infer statements from known statements and RDFS/OWL-Schematas.

It uses the same interface as MemModel, thus making the infererence process hidden.   
  
History:

- 09-23-2004 : Bug removed in \_findRuleEntailmentInIndex().
- 09-15-2004 : Added Index over InRule Triggers
  and Entailments to increase performance.
- 09-10-2004 : First version of this class.

- access: public
- author: Daniel Westphal <at d-westphal dot de mail at d-westphal dot de>
- version: V0.9.1

Located in /infModel/InfModel.php (line 28)

```
Object
   |
   --Model
      |
      --MemModel
         |
         --InfModel
```

Direct descendents

Description |
Descendents
| Methods (details)

| Class | Description |
| --- | --- |
| InfModelB | A InfModelB extends the InfModel Class, with a backward chaining algorithm. |
| InfModelF | A InfModelF extends the InfModel Class, with a forward chaining algorithm. |

Method Summary

Description |
Descendents |
Methods (details)

InfModel
InfModel
([string $baseURI = NULL])

void
add
(object Statement $statement)

object MemModel
findRegex
(string $subject\_regex, string $predicate\_regex, string $object\_regex)

object MemModel
findVocabulary
(string $vocabulary)

object Model
getSchema
()

void
replace
(object Node $subject, object Node $predicate, object Node $object, object Node $replacement)

Methods

Description |
Descendents |
Methods (details)

Constructor InfModel (line 82)

Constructor You can supply a base\_uri

- access: public

InfModel

InfModel
([string $baseURI = NULL])

- string
  $baseURI

add (line 176)

Adds a new triple to the Model without checking if the statement is already in the Model.

So if you want a duplicate free MemModel use the addWithoutDuplicates() function (which is slower then add()) If the statement's predicate label is supported by the inference, the matching rules are added.

- throws: PhpError
- access: public

void

add
(object Statement $statement)

- object Statement
  $statement

---

Redefinition of:

MemModel::add()
:   Adds a new triple to the MemModel without checking if the statement is already in the MemModel.

---

Redefined in descendants as:

- InfModelB::add()
  : Adds a new triple to the Model without checking, if the statement is already in the Model. So if you want a duplicate free Model use the addWithoutDuplicates() function (which is slower then add())
- InfModelF::add()
  : Adds a new triple to the MemModel without checking if the statement is already in the MemModel.

findRegex (line 451)

Method to search for triples using Perl-style regular expressions.

NULL input for any parameter will match anything. Example: $result = $m->find\_regex( NULL, NULL, $regex ); Finds all triples where the label of the object node matches the regular expression. Returns an empty MemModel if nothing is found.

This function is disabled in the Inference Model

- access: public

object MemModel

findRegex
(string $subject\_regex, string $predicate\_regex, string $object\_regex)

- string
  $subject\_regex
- string
  $predicate\_regex
- string
  $object\_regex

---

Redefinition of:

MemModel::findRegex()
:   Method to search for triples using Perl-style regular expressions.

findVocabulary (line 471)

Returns all tripels of a certain vocabulary.

$vocabulary is the namespace of the vocabulary inluding a # : / char at the end. e.g. http://www.w3.org/2000/01/rdf-schema# Returns an empty MemModel if nothing is found.

This function is disabled in the Inference Model

- access: public

object MemModel

findVocabulary
(string $vocabulary)

- string
  $vocabulary

---

Redefinition of:

MemModel::findVocabulary()
:   Returns all tripels of a certain vocabulary.

getSchema (line 404)

Returns a model, containing all Statements, having a Predicate, that is supported by the inference.

- access: public

object Model

getSchema
()

replace (line 428)

General method to replace nodes of a MemModel.

This function is disabled in the Inference Model

- throws: PhpError
- access: public

void

replace
(object Node $subject, object Node $predicate, object Node $object, object Node $replacement)

- object Node
  $subject
- object Node
  $predicate
- object Node
  $object
- object Node
  $replacement

---

Redefinition of:

MemModel::replace()
:   General method to replace nodes of a MemModel.

#### Inherited Methods


Inherited From MemModel

> MemModel::MemModel()  
> MemModel::add()  
> MemModel::addModel()  
> MemModel::addNamespace()  
> MemModel::addParsedNamespaces()  
> MemModel::addWithoutDuplicates()  
> MemModel::close()  
> MemModel::contains()  
> MemModel::containsAll()  
> MemModel::containsAny()  
> MemModel::equals()  
> MemModel::find()  
> MemModel::findAsIterator()  
> MemModel::findCount()  
> MemModel::findFirstMatchingStatement()  
> MemModel::findRegex()  
> MemModel::findVocabulary()  
> MemModel::getIndexType()  
> MemModel::getParsedNamespaces()  
> MemModel::getStatementIterator()  
> MemModel::index()  
> MemModel::intersect()  
> MemModel::isEmpty()  
> MemModel::isIndexed()  
> MemModel::iterFind()  
> MemModel::rdqlQuery()  
> MemModel::rdqlQueryAsIterator()  
> MemModel::reify()  
> MemModel::remove()  
> MemModel::removeNamespace()  
> MemModel::replace()  
> MemModel::saveAs()  
> MemModel::setBaseURI()  
> MemModel::size()  
> MemModel::subtract()  
> MemModel::toString()  
> MemModel::toStringIncludingTriples()  
> MemModel::unite()  
> MemModel::writeAsHtml()  
> MemModel::writeAsHtmlTable()  
> MemModel::writeRdfToString()

Inherited From Model

> Model::Model()  
> Model::findForward()  
> Model::getBaseURI()  
> Model::getMemModelByRDQL()  
> Model::getOntModel()  
> Model::getResmodel()  
> Model::load()  
> Model::visualize()

Inherited From Object

> Object::toString()

Documentation generated on Thu, 7 Jul 2005 13:41:35 +0200 by phpDocumentor 1.3.0RC3
